# Activation of drought tolerant traits in crops: endophytes as elicitors

**DOI:** 10.1080/15592324.2022.2120300

**Published:** 2022-11-14

**Authors:** Karaba N Nataraja, KH Dhanyalakshmi, Geetha Govind, Ralf Oelmüller

**Affiliations:** a Plant Molecular Biology Laboratory, Department of Crop Physiology, University of Agricultural Sciences Bangalore (UASB), GKVK Bengaluru, India; b Department of Plant Physiology, Regional Agricultural Research Station Pattambi, Kerala Agricultural University, Thrissur, Kerala, India; c Department of Biotechnology, College of Agriculture, UASB, Hassan, India; d Department of Plant Physiology, Matthias Schleiden Institute of Genetics, Bioinformatics and Molecular, Botany, Friedrich-Schiller-University Jena, Jena, Germany

**Keywords:** Drought, endophytes, elicitors, adaptive traits

## Abstract

Drought challenges crop production worldwide. The issue is aggravated by frequent drought episodes and unpredictable rainfall patterns associated with global climate change. While the efforts to breed drought-resistant crop varieties are progressing, the need of the hour is immediate strategies to sustain the yields of existing ones. As per recent studies, stress adaptive traits can be activated using specific elicitors. Endophytes that inhabit host plants asymptomatically are natural elicitors/bio-stimulators capable of activating host gene expression, conferring several benefits to the hosts. This review discusses the scope of using trait-specific endophytes in activating drought adaptive traits in crop varieties.

## Introduction

Drought is one of the major limitations of crop production. The impact of drought on crop yields has been significant in the past,^1,2^ and the yield loss risk is aggravating further under the fast-changing climate scenario.^3^ Hence, to sustain productivity, there is a pressing need to build crop resilience toward drought under the changing climate. There has been intense research in this direction to enhance crop yields under water-limiting conditions through conventional, marker-assisted and transgenic approaches.^4,5,6,7^ However, there is an immense gap in achieving the research targets due to multiple factors, including the complex regulation of the drought tolerance traits at the molecular level, its manifestation at the field level as influenced by the external environment, biosafety concerns etc. This calls for rapid and eco-friendly interventions that can empower the crops with stress adaptive traits.

## The potential of endophytes in manipulating plant traits

It is now established that a plant phenotype is regulated by its associated microbiome. Thus, endophytes (fungal and bacterial) colonizing the plants asymptomatically and maintaining a symbiotic association can manipulate the host phenotype. Moreover, this association has shown to be greatly advantageous to the host plants, conferring numerous health benefits such as growth promotion through the accumulation of phytohormones,^8^ improved nutrient acquisition,^9^ and tolerance to biotic and abiotic stresses [Reviewed in ^10^]. It is also demonstrated that endophytes prospected from the plants inhabiting harsh environments can extend the benefits of habitat-adapted symbiosis to non-host crop systems, thereby improving the host performance under challenging conditions.^11,12^ On these grounds, endophytes have been considered to be an eco-friendly alternative to manipulate drought tolerance traits in crop plants [Reviews 10,13].

## Mechanisms governing endophyte-mediated management of drought tolerance traits

Studies from diverse crop systems indicate that endophytes can trigger a wide array of molecular morpho-physiological and biochemical responses (including photosynthesis) that improve the host plant’s resistance to drought [Reviewed in ^13^]. Many studies demonstrate that endophyte enrichment improves overall plant growth under drought, primarily achieved by improving the root biomass,^14^ enhancing the uptake of nutrients like phosphorus and zinc,^15^ the protection of photosynthetic machinery and enhancing photosynthesis.^16^ This can be either due to the direct effect of the endophytes on plant growth enacted by the secretion of phytohormones, bioactive compounds etc.,^17^ or indirectly by altering the host gene expression and physiology,^12^ which ultimately target plant water relations (uptake and conservation), and activate cellular level tolerance traits.

Many auxin-producing endophytes are known to directly promote root growth in host plants under water-limiting conditions.^18^ Several endophytes function by activating auxin biosynthesis in their host leading to the modification of the root architecture through increasing root length, number, volume and biomass, thereby improving the water mining ability of the host under drought.^19^ While abscisic acid (ABA) secreted by the endophytes functions as an elicitor to activate a signaling cascade resulting in drought resistance responses in the host^20^, several others modulate drought responses by increasing the ABA concentration within the host.^21^ Endophytes targeting stomatal traits (lower stomatal conductance and stomatal density) contribute to improved water use efficiency under drought.^21^ Water conservation is also achieved by modifying the cuticular wax load on the host, an important trait for drought adaptation. Here, endophyte inoculation significantly altered the expression of the wax biosynthetic pathway genes (for example, FabG, desB, SSI2, fadD, BiP, KCS, KAR, FAR, and ABCB1) under drought, resulting in an altered wax composition in the host.^22^ Many studies demonstrate the role of endophytes in conferring drought tolerance by activating diverse cellular level tolerance traits. Increased reactive oxygen species scavenging capacity^23^ under drought minimizes the damage to cellular membranes and macromolecules. Endophyte-induced accumulation of compatible solutes like proline, glycine betaine, soluble sugars and organic acids,^24,20^ contributes to drought tolerance via lowering osmotic potential and turgor maintenance. Activation of the transcription of drought-responsive genes in the host is another important mechanism underlying endophyte-mediated drought tolerance.^25^ Expression levels of the native stress-responsive genes (ANAC072, DREB2A, CIPK3, ERD1, CBL1, HAT, PLDδ), whose products are linked with diverse signaling, protection and metabolic functions in the cell, are significantly increased in drought-stressed maize, in the presence of endophytes.^26^ Another example is the activation of the proline biosynthetic pathway gene (P5CS) in the presence of endophyte, which contributes to enhanced proline synthesis under drought.^15^ It is showed that endophytes trigger reprogramming of the transcriptome,^27^ proteome and metabolome,^28^ leading to the activation of stress adaptive traits in host plants.

## The choice of endophytes for trait activation

For better and rapid results, endophytes from habitat-adapted plants closely related to the choice crop are to be prospected. Endophytes from related species or wild relatives must be encouraged to avoid off-target responses. The focus must be on manipulating specific/ multiple drought adaptive traits in a broad host range for commercial applications.^29^ Endophytes that are plant growth stage-, tissue- and growth condition-specific might yield better results without a trade-off on carbon use. Moreover, the mechanisms by which a selected endophyte confers drought tolerance in target hosts must be characterized for better utilization. The application of endophyte consortia has been yielding promising results in various crops.^30^ Still, it demands more research on their interaction with each other within the plant and with the external environment. Since there are reports on the transfer of endophytes from parents to offspring,^31^ the approach of endophyte enrichment would be highly durable. As the conventional breeding programs focused on plant genomes as a unit of selection, there is a need to evolve programs wherein holobiont is the unit of selection,^32^ with adequate strategies for characterization of the endophyte enriched plant types. The success in eliciting the responses under field conditions depends on the precise understanding of the underlying molecular mechanisms mediated by endophyte-host genomes, and there must be a concerted effort in this direction.

## Conclusion

Water is already a limiting factor for crop production. Endophyte-mediated activation of drought tolerance traits would be an eco-friendly and sustainable method of conferring drought tolerance in existing crop varieties. Moreover, in nature, drought is always associated with other abiotic/combined stress conditions such as high temperature, osmotic stress etc. Endophyte enrichment can confer tolerance to multiple stress conditions at a time, and activation of basic physiological processes like photosynthesis, as reported in many studies, would help sustain productivity under stressful conditions. However, it requires extensive characterization of the type of endophyte that can be used for a specific crop, its interaction with the host holobiome, and the performance of the altered holobiont under drought. Research in this direction would be promising as an immediate alternative to enhance drought tolerance in crop varieties.

## References

[1] Daryanto S, Wang L, Jacinthe PA. Global synthesis of drought effects on food legume production. PloS One. 2015;10(6):e0127401. doi:10.1371/journal.pone.0127401. 26061704 PMC4464651

[2] Lesk C, Rowhani P, Ramankutty N. Influence of extreme weather disasters on global crop production. Nature. 2016;529(7584):84–3. doi:10.1038/nature16467. 26738594

[3] Leng G, Hall J. Crop yield sensitivity of global major agricultural countries to droughts and the projected changes in the future. Sci Total Environ. 2019;654:811–821. doi:10.1016/j.scitotenv.2018.10.434. 30448671 PMC6341212

[4] Karaba A, Dixit S, Greco R, Aharoni A, Trijatmiko KR, Marsch-Martinez N, Krishnan A, Nataraja KN, Udayakumar M, Pereira A. 2007. Improvement of water use efficiency in rice by expression of HARDY, an Arabidopsis drought and salt tolerance gene. Proceedings of the National Academy of Sciences, 104(39), pp.15270–15275. 10.1073/pnas.0707294104 PMC1986572 17881564

[5] Sajeevan RS, Nataraja KN, Shivashankara KS, Pallavi N, Gurumurthy DS, Shivanna MB. Expression of Arabidopsis SHN1 in Indian mulberry (*Morus indica L.*) increases leaf surface wax content and reduces post-harvest water loss. Front Plant Sci. 2017;8:418. doi:10.3389/fpls.2017.00418. 28421085 PMC5378817

[6] González FG, Capella M, Ribichich KF, Curín F, Giacomelli JI, Ayala F, Watson G, Otegui ME, Chan RL. Field-grown transgenic wheat expressing the sunflower gene HaHB4 significantly outyields the wild type. J Exp Bot. 2019;70(5):1669–1681. doi:10.1093/jxb/erz037. 30726944 PMC6411379

[7] Bharadwaj C, Tripathi S, Soren KR, Thudi M, Singh RK, Sheoran S, Roorkiwal M, Patil BS, Chitikineni A, Palakurthi R, et al. Introgression of “QTL‐hotspot” region enhances drought tolerance and grain yield in three elite chickpea cultivars. Plant Genome. 2021;14(1):e20076. doi:10.1002/tpg2.20076. 33480153 PMC12807433

[8] Bilal L, Asaf S, Hamayun M, Gul H, Iqbal A, Ullah I, Lee IJ, Hussain A. Plant growth promoting endophytic fungi *Asprgillus fumigatus* TS1 and *Fusarium proliferatum* BRL1 produce gibberellins and regulates plant endogenous hormones. Symbiosis. 2018;76(2):117–127. doi:10.1007/s13199-018-0545-4.

[9] Varga T, Hixson KK, Ahkami AH, Sher AW, Barnes ME, Chu RK, Battu AK, Nicora CD, Winkler TE, Reno LR, et al. Endophyte-promoted phosphorus solubilization in populus. Front Plant Sci. 2020;1585. 10.3389/fpls.2020.567918 PMC7609660 33193494

[10] Chitnis VR, Suryanarayanan TS, Nataraja KN, Prasad SR, Oelmüller R, Shaanker RU. Fungal endophyte-mediated crop improvement: the way ahead. Front Plant Sci. 2020;11:1588. doi:10.3389/fpls.2020.561007. PMC7652991 33193487

[11] Sangamesh MB, Jambagi S, Vasanthakumari MM, Shetty NJ, Kolte H, Ravikanth G, Nataraja KN, Uma Shaanker R. Thermotolerance of fungal endophytes isolated from plants adapted to the Thar Desert, India. Symbiosis. 2018;75(2):135–147. doi:10.1007/s13199-017-0527-y.

[12] Molina-Montenegro MA, Acuña-Rodríguez IS, Torres-Díaz C, Gundel PE, Dreyer I. Antarctic root endophytes improve physiological performance and yield in crops under salt stress by enhanced energy production and Na^+^ sequestration. Sci Rep. 2020;10(1):1–10. doi:10.1038/s41598-020-62544-4. 32242034 PMC7118072

[13] Verma H, Kumar D, Kumar V, Kumari M, Singh SK, Sharma VK, Droby S, Santoyo G, White JF, Kumar A. The potential application of endophytes in management of stress from drought and salinity in crop plants. Microorganisms. 2021;9(8):1729. doi:10.3390/microorganisms9081729. 34442808 PMC8398416

[14] Li X, He C, He X, Su F, Hou L, Ren Y, Hou Y. Dark septate endophytes improve the growth of host and non-host plants under drought stress through altered root development. Plant Soil. 2019;439(1):259–272. doi:10.1007/s11104-019-04057-2.

[15] Saddique MAB, Ali Z, Khan AS, Rana IA, Shamsi IH. Inoculation with the endophyte *Piriformospora indica* significantly affects mechanisms involved in osmotic stress in rice. Rice. 2018;11(1):1–12. doi:10.1186/s12284-018-0226-1. 29799607 PMC5968016

[16] Zhang W, Xie Z, Zhang X, Lang D, Zhang X. Growth-promoting bacteria alleviates drought stress of G. uralensis through improving photosynthesis characteristics and water status. J Plant Interact. 2019;14(1):580–589. doi:10.1080/17429145.2019.1680752.

[17] Waqas M, Khan AL, Kamran M, Hamayun M, Kang SM, Kim YH, Lee IJ. Endophytic fungi produce gibberellins and indoleacetic acid and promotes host-plant growth during stress. Molecules. 2012;17(9):10754–10773. doi:10.3390/molecules170910754. 22960869 PMC6268353

[18] Qiang X, Ding J, Lin W, Li Q, Xu C, Zheng Q, Li Y. Alleviation of the detrimental effect of water deficit on wheat (*Triticum aestivum* L.) growth by an indole acetic acid-producing endophytic fungus. Plant Soil. 2019;439(1):373–391. doi:10.1007/s11104-019-04028-7.

[19] Liu Y, Wei X. Dark septate endophyte improves drought tolerance of *Ormosia hosiei* Hemsley & EH Wilson by modulating root morphology, ultrastructure, and the ratio of root hormones. Forests. 2019;10(10):830. doi:10.3390/f10100830.

[20] Zhou, X.R., Dai, L., Xu, G.F. and Wang, H.S. A strain of Phoma species improves drought tolerance of *Pinus tabulaeformis*. A strain of Phoma species improves drought tolerance of Pinus tabulaeformis. 2021;11(1), pp.1-11. 10.1038/s41598-021-87105-1 PMC8027514 33828138

[21] Rho H, Van Epps V, Wegley N, Doty SL, Kim SH. Salicaceae endophytes modulate stomatal behavior and increase water use efficiency in rice. Front Plant Sci. 2018a;9:188. doi:10.3389/fpls.2018.00188. 29552021 PMC5840156

[22] Zhao Z, Ju Y, Kou M, Tian M, Christensen MJ, Zhang X, Nan Z. Cuticular wax modification by epichloë endophyte in *Achnatherum inebrians* under different soil moisture availability. J Fungi. 2022;8(7):725. doi:10.3390/jof8070725. PMC9325231 35887480

[23] Tsai HJ, Shao KH, Chan MT, Cheng CP, Yeh KW, Oelmüller R, Wang SJ. *Piriformospora indica* symbiosis improves water stress tolerance of rice through regulating stomata behavior and ROS scavenging systems. Plant Signal Behav. 2020;15(2):1722447. doi:10.1080/15592324.2020.1722447. 32024420 PMC7053885

[24] Nagabhyru P, Dinkins RD, Wood CL, Bacon CW, Schardl CL. Tall fescue endophyte effects on tolerance to water-deficit stress. BMC Plant Biol. 2013;13(1):1–17. doi:10.1186/1471-2229-13-127. 24015904 PMC3848598

[25] Sherameti I, Tripathi S, Varma A, Oelmüller R. The root-colonizing endophyte *Pirifomospora indica* confers drought tolerance in Arabidopsis by stimulating the expression of drought stress–related genes in leaves. Mol Plant-Microbe Interact. 2008;21(6):799–807. doi:10.1094/MPMI-21-6-0799. 18624643

[26] Xu L, Wang A, Wang J, Wei Q, Zhang W. *Piriformospora indica confers drought tolerance on Zea mays* L. through enhancement of antioxidant activity and expression of drought-related genes. Crop J. 2017;5(3):251–258. doi:10.1016/j.cj.2016.10.002.

[27] Sampangi-Ramaiah MH, Dey P, Jambagi S, Vasantha Kumari MM, Oelmüller R, Nataraja KN, Venkataramana Ravishankar K, Ravikanth G, Uma Shaanker R. An endophyte from salt-adapted Pokkali rice confers salt tolerance to a salt-sensitive rice variety and targets a unique pattern of genes in its new host. Sci Rep. 2020;10(1):1–14. doi:10.1038/s41598-020-59998-x. 32094443 PMC7039991

[28] Ghaffari MR, Mirzaei M, Ghabooli M, Khatabi B, Wu Y, Zabet-Moghaddam M, Mohammadi-Nejad G, Haynes PA, Hajirezaei MR, Sepehri M, et al. Root endophytic fungus *Piriformospora indica* improves drought stress adaptation in barley by metabolic and proteomic reprogramming. Environ Exp Bot. 2019;157:197–210. doi:10.1016/j.envexpbot.2018.10.002.

[29] Dhanyalakshmi KH, Mounashree DC, Vidyashree DN, Earanna N, Nataraja KN. Options and opportunities for manipulation of drought traits using endophytes in crops. Plant Physiol Rep. 2019;24(4):555–562. doi:10.1007/s40502-019-00485-5.

[30] Rho H, Hsieh M, Kandel SL, Cantillo J, Doty SL, Kim SH. Do endophytes promote growth of host plants under stress? A meta-analysis on plant stress mitigation by endophytes. Microb Ecol. 2018b;75(2):407–418. doi:10.1007/s00248-017-1054-3. 28840330

[31] Vandenkoornhuyse P, Quaiser A, Duhamel M, Le Van A, Dufresne A. The importance of the microbiome of the plant holobiont. New Phytologist. 2015;206(4):1196–1206. doi:10.1111/nph.13312. 25655016

[32] Gopal M, Gupta A. Microbiome selection could spur next-generation plant breeding strategies. Front Microbiol. 2016;7:1971. doi:10.3389/fmicb.2016.01971. 28003808 PMC5141590

